# Biomonitoring of Polycyclic Aromatic Hydrocarbon Deposition in Greenland Using Historical Moss Herbarium Specimens Shows a Decrease in Pollution During the 20^th^ Century

**DOI:** 10.3389/fpls.2020.01085

**Published:** 2020-07-15

**Authors:** Karen Martinez-Swatson, Eszter Mihály, Christian Lange, Madeleine Ernst, Majbrit Dela Cruz, Michelle J. Price, Teis Nørgaard Mikkelsen, Jan H. Christensen, Nina Lundholm, Nina Rønsted

**Affiliations:** ^1^ Natural History Museum of Denmark, Faculty of Science, University of Copenhagen, Copenhagen, Denmark; ^2^ Center for Newborn Screening, Department of Congenital Disorders, Statens Serum Institute, Copenhagen, Denmark; ^3^ Department of Plant and Environmental Sciences, Faculty of Science, University of Copenhagen, Frederiksberg, Denmark; ^4^ Conservatoire et Jardin Botaniques de la Ville de Genève, Geneva, Switzerland; ^5^ Department of Environmental Engineering, Technical University of Denmark, Lyngby, Denmark; ^6^ Science and Conservation, National Tropical Botanical Garden, Kalaheo, HI, United States

**Keywords:** atmospheric pollution, bioindicators, bryophytes, Gas Chromatography - Mass Spectrometry (GC-MS), herbaria, historical trends, Polycyclic Aromatic Hydrocarbons (PAHs), Persistent Organic Pollutants (POPs)

## Abstract

Although most point sources of persistent organic pollutants (POPs), including polycyclic aromatic hydrocarbons (PAHs), are at lower latitudes, the Arctic region is contaminated. In particular, PAHs now dominate the POP body burden of the region’s marine biota at the lower trophic levels. Greenlandic Inuits have the most elevated levels of POPs in their blood compared to any other population, due to their consumption of seal meat and other marine mammals. PAHs, the by-products of the incomplete combustion of petroleum products, are known carcinogens and have been shown to affect the immune system, reproduction, endocrine functions, and the nervous system. With industrial activities and climate change set to increase local PAH emissions, it is paramount to document changes in atmospheric PAH deposition to further investigate PAH exposure in the region and attribute contaminations to their sources. As a measure of atmospheric pollution, we sampled bryophyte herbarium specimens of three common and widespread species collected in Greenland between the 1920s and 1970s after which time new collections were not available. They were analyzed for 19 PAHs using GC-MS (gas chromatography mass spectrometry). The presence of more low-molecular-weight PAHs than high-molecular-weight PAHs is evidence that the PAH contamination in Greenland is due to long-range transport rather than originating from local sources. The results show peaks in PAH atmospheric deposition in the first part of the 19th century followed by a trend of decrease, which mirror global trends in atmospheric pollution known from those periods. PAHs associated with wood and fossil-fuel combustion decrease in the 1970s coinciding with the disappearance of charcoal pits and foundries in Europe and North America, and a shift away from domestic heating with wood during the 19^th^ century. The results highlight the value of bryophytes as bioindicators to measure PAH atmospheric pollution as well as the unrealized potential of herbaria as historical records of environmental change.

## Introduction

Persistent organic pollutants (POPs) comprise toxic and bio-accumulative compounds, that are introduced into the environment through anthropogenic activities since the 1900s (Bengtson Nash, 2011). They are used globally in agricultural, industrial and health applications (Corsolini et al., 2016). Due to their effective environmental dispersal mechanisms and their resistance to degradation, they have contaminated all ecosystems globally, and they pose a substantial risk to human health and the environment (Bengtson Nash, 2011). As their distribution is wide-ranging and across national borders, there has been international cooperation to stop or substantially reduce the release of a growing number of these chemicals. Most notably in the form of the Stockholm Convention, first adopted in 2001, to protect human health and the environment from POPs and signed by 182 countries (UNEP, 2009; UNEP, 2013). Although global restrictions have led to a reduction in the release of POPs, their levels in the environment and within animal populations still remain high enough to elicit concern (Sonne, 2010; Bengtson Nash, 2011; AMAP, 2018). Exposure to POPs in humans and animals has been shown to increase the risk of adverse effects on the immune system (Corsini et al., 2014; Oulhote et al., 2017), reproduction (Vested et al., 2014; Dietz et al., 2018), endocrine functions (Hotchkiss et al., 2008; Berg et al., 2016; Ahmed et al., 2019), the nervous system (Dingemans et al., 2011), and to cause cancer (Alavanja and Bonner, 2012), among others.

Although most sources of POPs originate at lower latitudes, the Arctic region is known to be contaminated. The main transport mechanism for POPs is long-range atmospheric transport (Wania, 2003). POPs are known to deposit out of the atmosphere according to their volatility, along latitudinal and altitudinal temperature gradients (Bengtson Nash, 2011). They can be subjected to global distillation whereby they are repeatedly volatilised and condensed in and out of the atmosphere. These repeated cycles of volatilisation and deposition, exacerbated by the prolonged half-lives of these compounds, result in their progressive movement from their point of origin in temperate and tropical regions towards colder climates. Furthermore, “cold trapping” at high altitudes or at the Earth’s poles further contributes to their accumulation and prolonged persistence in these regions, as they are not able to re-enter the atmosphere (AMAP, 2004; Bengtson Nash, 2011). This therefore makes cold regions like the Arctic a sink for POPs. Northern flowing ocean currents or rivers have also been shown to transport water-soluble POPs from industrial regions in the Northern hemisphere and up to the Arctic, as have migratory birds to a lesser extent (AMAP, 2004). In addition, concern has been raised that despite a decline in these chemicals due to their global regulation, Arctic warming and ice retreat due to climate change, is causing their release into the atmosphere from sinks such as water, snow, ice, and soils (Ma et al., 2011).

**Figure 1:**
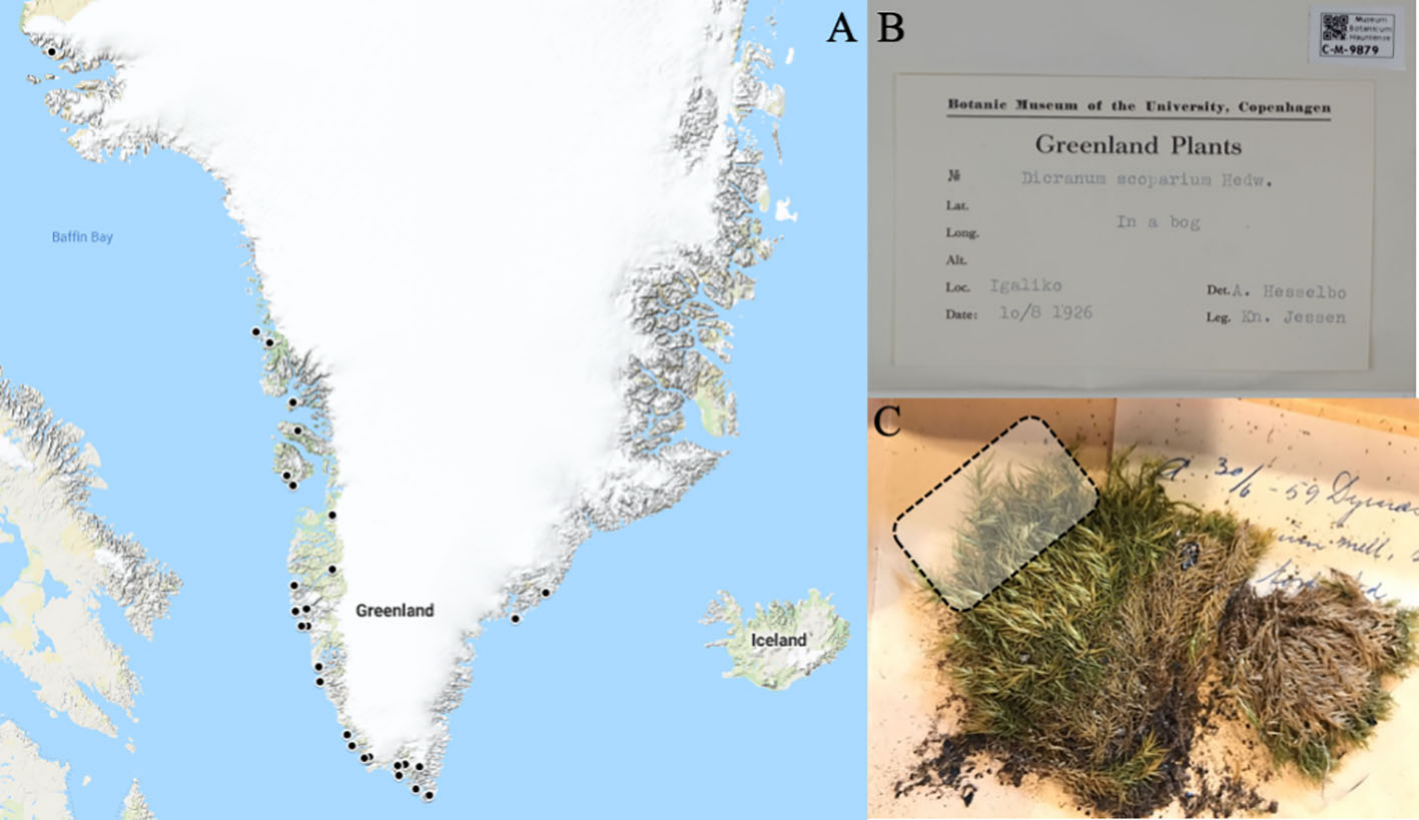
**(A)** Map of collection sites in Greenland where herbarium specimens were sampled, for vouchers with enough information on locality (localities marked by•). **(B)** Example of voucher specimen (C-M-9879). **(C)** *Dicranum scoparium* herbarium specimen (C-M-9879) with an indication of the part sampled from the voucher illustrated by dotted square.

Polycyclic aromatic hydrocarbons (PAHs) are a class of POPs composed of carbon and hydrogen with two or more benzene rings. They are semi-volatile, lipophilic, and resistant to heat and corrosion (Masih et al., 2012; Das et al., 2014). They can be divided into two categories: low molecular weight compounds (with less than four benzene rings) and high molecular weight compounds (with four or more benzene rings) (Kim et al., 2013) (**Figure 1**). They were one of the first groups of atmospheric pollutants to be identified as carcinogenic, with carcinogenicity increasing with the molecular weight of the compound (Ames et al., 1972). PAHs are formed and emitted in large quantities as the by-products of the incomplete combustion of petroleum products like coal, fuel, and gas (Jiao et al., 2017). They are hydrophobic and difficult to degrade, which therefore leads to increased accumulation in soils. Their levels have been shown to be increasing within Arctic marine biota at lower trophic levels in contrast to other POPs that are in decline, and PAHs now dominate the POP body burden of Arctic invertebrates and fish (Laender et al., 2011). Like with other POPs, atmospheric transport is the main mechanism for PAHs produced at lower latitudes to find their way to Arctic regions.

The lipophilicity of many POPs, including PAHs, accelerates their accumulation in plant and animal tissues, magnifying in concentration up the food chain (Franzaring and van der Eerden, 2000). Furthermore, they are metabolized in animal tissue, rendering them carcinogenic (Zhang et al., 2016). They usually enter the food web through the consumption of phytoplankton by organisms at lower trophic levels, which are then consumed by larger marine biota, accumulating POPs in their fatty tissues and increasing in concentration at each step of the trophic chain (Corsolini et al., 2016). These compounds are therefore detectable in the tissues of many high trophic-level predators of the Arctic marine ecosystem, such as the Greenland polar bear (*Ursus maritimus* Phipps) and the Greenland sleeper shark (*Somniosus microcephalus* Bloch & J. G. Schneider) (Corsolini et al., 2016; Styrishave et al., 2017). Much higher levels of POP contamination have also been found in humans living in the Arctic regions compared with people living in other regions, which is due to a largely marine based diet. In particular, the Greenlandic Inuit population, who consume seal meat and other marine mammals, have been under close scrutiny, as they have been found to have highly elevated levels of POPs in their blood (Oostdam and Tremblay, 2002).

Greenland’s economy is largely based on fishing with a small number of mining projects (Statistics Greenland, 2018). Greenland has the lowest human population density in the world, with its population confined mainly to the south-western coast (Statistics Greenland, 2018). Eighty-one percent of the country is covered by an ice sheet that extends over 2,500 km from the North to the South and around 1,000 km from East to West (Statistics Greenland, 2018; Brugger et al., 2019). Due to its isolation, small population and lack of industry, Greenland was long thought to be a pristine environment. However, industrial emissions have led to the deposition of atmospheric pollutants across the region since the 1850s (McConnell et al., 2007). Combined with the cold climate trapping them in snow and ice, Greenland has become a sink for pollutants. They are transported to Greenland from a very wide area, including the North Atlantic, North America, and central Asia (Schüpbach et al., 2018). With industrial activities (oil, gas, and mining) causing local PAH emissions to increase in the region, as well as climate change contributing to changes in the transport and re-volatilisation of PAHs (Christiansen et al., 2014; Friedman et al., 2014; Tiainen, 2016), it is therefore important to monitor and document changes in atmospheric PAH concentrations and exposure, as well as to attribute the contaminants to their sources.

Greenland’s landscape is mostly tundra with an arctic plant diversity largely comprised of bryophytes (Farge et al., 2013). The quality of the environment and how it changes over time can be assessed using so-called “bio-indicators”, comprising certain species or groups of species and in some cases biological processes that are analyzed qualitatively. Biomonitors are organisms or communities from which quantitative measures for the assessment of environmental quality are obtained (Markert, 2007). The basic criteria for organisms to be used as biomonitors of atmospheric pollution are that: they readily accumulate airborne pollutants in their tissues, the levels of pollutants in their tissues vary based on the distance from the pollution source, the natural levels of pollutants in their tissues is stable, and they possess morphological/biological/physiological features that render them sensitive to significant changes in pollution levels. Bryophytes, with their morphological and eco-physiological particularities i.e. small, non-vascular plants with simple stems and leaves that demonstrate poikilohydry, e.g. the lack of structural or functional mechanisms to maintain and/or regulate water content, often coupled with desiccation tolerance (Proctor et al., 2007), have significant potential as biomonitors (Onianwa, 2001). Bryophytes have been used to measure atmospheric deposition of PAHs in a number of recent studies (Ötvös et al., 2004; Liu et al., 2005; Gałuszka, 2007; Krommer et al., 2007; Foan et al., 2010; Ciesielczuk et al., 2012; Çabuk et al., 2014; Foan et al., 2015; Oishi, 2018), but never in bryophyte rich Greenland.

Whereas modern moss samples can provide a measure of current day contamination, herbarium collections represent records, in time and space, of the world’s plants. Herbaria can therefore be very useful tools for environmental research, with habitat loss, biological invasions, and climate change being just some of the areas in which herbarium collections have recently been used (Suarez and Tsutsui, 2004). A previous study by Foan et al. (2010) has also shown the potential of moss herbarium specimens to determine long-term temporal changes in atmospheric PAH deposition. The Natural History Museum of Denmark at the University of Copenhagen (Herbarium C) houses the largest collection of plant specimens from Greenland in the world, and specifically of bryophytes as they are a dominant component of the vegetation, including collections assembled through regular expeditions to Greenland since the 17^th^ century collected by e.g. Poul Egede, Jens Vahl, Christian E. Otterstrøm Jensen, Kjeld Holmen, and Kell Damsholt among others (Holmen et al., 1974; Lewinsky, 1974; Gjærevoll and Ryvarden, 1977; Damsholt, 2013).

The present study therefore takes advantage of the historical bryophyte collections from Greenland that are housed in Herbarium C to gather samples for monitoring environmental contamination by POPs in Greenland and also to demonstrate the value of herbarium collections as historical records of anthropogenic effects on the environment. The objective of this study was thus to measure the concentration of PAHs in herbarium specimens of bryophytes from Greenland at different time points to investigate historical trends in atmospheric pollution in Greenland.

## Materials and Methods

### Herbarium Bryophyte Sampling

Three species were selected from the herbarium of the Natural History Museum of Denmark, University of Copenhagen (Herbarium C) - *Dicranum scoparium* Hedw., *Hylocomium splendens* (Hedw.) Schimp. and *Racomitrium lanuginosum* (Hedw.) Brid. These three species were selected as they represent widespread and common taxa in Greenland and across Europe (Smith, 2004), which have been used for bio-monitoring in past studies (Foan et al., 2010; Foan et al., 2015; Wojtuń et al., 2018) and were available in the herbarium across a continuous and comparable timespan. Unfortunately, bryophyte collection efforts in Greenland has been very limited after the 1970s and we were therefore not able to extend the study to the present day. Each of the three species was sampled in triplicate across five different time points, availability of herbarium material permitting (1920s, 1940s, 1950s, 1960s, and 1970s; see **Supplementary Table S1**). Only one sample was available from the 1930s (C-M 9925, 1932) and this sample was therefore included in the 1920s category. Furthermore, only 13 samples could be obtained for *D. scoparium*, giving a total of 42 samples (each >1g), from different localities in Greenland (**Figure 1**). Leaves from the upper stems were taken from the voucher specimens with metal tweezers (as shown in **Figure 1C**).

### Sample Preparation

For each sample, 0.38–1.64 g was homogenized with an equal amount of hydromatrix in an IKA 11® sample mill (IKA, USA). Samples were then extracted via pressurized liquid extraction in an ASE200 (Dionex, Denmark). The extraction cells were composed of 4 g activated silica in the bottom layer as a chlorophyll retainer, 2–5 g of sample mix depending on the weight of the sample, and a top layer of Ottawa sand. Two hundred µl of internal standard mix (8 µg ml^-1^, **Table S2**) was added to each sample. The following extraction parameters were used: pressure 1,500 psi, preheat time of 2 min, static time of 5 min, 70% flush volume, 60 s purge time, 2 static cycles, 100°C, n-pentane:dichloromethane (90:10) solvent mixture. Each cell was extracted twice into separate collection vials. After concentrating under 40°C, the two extracts were combined and evaporated to less than 5 ml. 200 µl of recovery standard mix (8µg ml^-1^) was added and the samples were reconstituted to 5 ml with n-pentane:dichloromethane (90:10).

### GC-MS Analysis

The moss extracts were analyzed for PAHs on an Agilent 7890A gas chromatograph (GC) with an Agilent 5975C inert XL mass spectrometer (MS) with electron ionisation, operating in selected ion monitoring (SIM) mode. The GC-MS parameters were as follows: 1 μl sample was injected in splitless mode (inlet: 300°C) to a 60 m HP-5 capillary column with 0.25 mm inner diameter, 0.25 μm film thickness. The flow rate was 1.1 ml min^-1^. The initial temperature of 40°C was held for 2 min, increased by 25°C min^-1^ to 100°C, followed by an increase of 5°C min^-1^ to 315°C and held for 14 min (total run time: 61.4 min). The temperatures of the transfer line, ion source, and quadrupole were 315, 230, and 150°C respectively.

### Data Processing and Analysis

Peaks were quantified using MassHunter Workstation (Quantitative Analysis Version B.07.00/Build 7.0.457.0 for GCMS, Agilent technologies, Inc.). PAH concentrations were calculated from a 6-point calibration curve using deuterated PAHs as internal and recovery standards. The concentration of each compound was calculated as ng g^-1^ dry weight of sample. Only values above the limit of quantification (LOQ) were retained and LOQ values are listed in **Table S3**).

Data handling, visualization, and analysis was performed in the statistical software package R (R Core Team, 2016) with the add-on packages ggplot2 (Wickham, 2016), gridExtra (Auguie, 2017), multcompView (Graves et al., 2015), RColorBrewer (Neuwirth, 2014), scales (Wickham, 2018), superheat (Barter and Yu, 2017), plyr (Wickham, 2011), and vegan (Oksanen et al., 2019). To visually assess patterns in PAHs across decades and species, we created a heat map.

Subsequently, to assess whether there is a statistically significant difference in mean PAH concentrations across decades or industrial period, we performed an analysis of variance (ANOVA), combined with a Tukey’s honest significance test. While an ANOVA tests for whether mean PAH concentrations are significantly different across decades or industrial period, the Tukey’s honest significance test performs pair-wise comparisons of all means and identifies pairs, which are significantly different to each other. P-values of the ANOVA were corrected for multiple hypotheses testing using the false-discovery rate (FDR) method (Benjamini and Hochberg, 1995). Prior to statistical analysis, all values below LOQ were replaced with 0, and only PAHs with at least 20 out of 42 samples with non-zero values were taken into consideration, corresponding to naphthalene, phenanthrene, fluoranthene, and pyrene. To make our data conform to the normal distribution, we performed a log transformation.

Finally, to test how chemically dissimilar bryophytes are across different decades, we assessed pair-wise chemical dissimilarity using the Bray-Curtis dissimilarity metric followed by a Tukey’s honest significance test. The R script used for data handling, visualisation and analysis is publicly available as a Jupyter notebook at https://github.com/madeleineernst.

## Results

A total of 19 PAHs were measured in this study (see **Table S3**). Of these, 11 were detected in our samples (full dataset in **Figure S1**, **Table S4**). The means and ranges of the PAHs (ng g^-1^ dry weight) across the time periods sampled are shown in **Table 1**. The highest concentration was observed for phenanthrene for the 1920s with 1252.9 ng g^-1^. This was followed by naphthalene with a concentration of 897.0 ng g^-1^ in the 1940s, fluoranthene with 191.8 ng g^-1^ in the 1920s, and pyrene with 114.7 ng g^-1^ in the 1940s.

**Table 1:** The means and ranges of polycyclic aromatic hydrocarbon (PAH) concentrations (ng g^-1^ dry weight) found in three species of bryophyte from the Greenland herbarium collection in C for different sampling periods from the 1920s to 1970s.

PAHs (means & observed ranges ng g^-1^)	IARC carcinogenicity classification	Sampling Period				
		1920s	1940s	1950s	1960s	1970s
Naphthalene	2B	370.6 163.9-484.9	304.1 <LOQ-671.8	221.5 <LOQ-417.2	116.5 <LOQ-208.0	231.2 <LOQ-379.2
Phenanthrene		258.6 71.5-221.4	134.6 <LOQ-278.92	127.7 <LOQ-138.6	286.6 <LOQ-1252.9	115.9 <LOQ-221.4
Fluoranthene		80.4 25.5-143.8	47.2 12.7-98.4	43.2 <LOQ-165.5	57.8 9.2-191.8	36.2 9.2-95.3
Pyrene		55.0 19.8-97.7	33.7 9.2-71.8	33.7 <LOQ-114.7	40.7 <LOQ-112.9	24.9 7.0-49.1
Benzo[a]anthracene	2B	9.8 <LOQ-10.2	13.1 <LOQ-15.2	17.3 <LOQ-20.8	6.3 <LOQ-7.2	<LOQ
Chrysene	2B	41.0 <LOQ-44.8	<LOQ	42.4 <LOQ-49.5	<LOQ	<LOQ-35.4
Benzo[b]fluoranthene	2B	<LOQ	<LOQ	<LOQ -46.3	<LOQ	<LOQ
Benzo[k]fluoranthene	2B	<LOQ	<LOQ	<LOQ-50.7	<LOQ	<LOQ
Benzo[ghi]perylene		<LOQ	<LOQ-18.9	<LOQ-37.2	<LOQ	<LOQ
LMW PAHs		709.6 351.8-1445.9	422.3 13.9-1073.1	314.9 17.7-772.8	390.0 65.2-1567.1	216.2 9.3-675.4
HMW PAHs		71.9 19.8-110.9	38.7 9.2-105.9	52.3 6.8-319.3	38.0 7.1-119.2	24.3 7.0-74.6
ΣPAHs		781.6 387.8-1590.3	461.0 25.3-1176.5	367.3 30.1-1092.1	428.0 72.3-1686.3	240.5 16.2-724.6

The table includes individual PAH concentrations and PAH concentrations for all high molecular weight (HMW) PAHs, low molecular weight (LMW) PAHs and total PAH concentration (ΣPAHs) for each sampling period. Means are only given for PAHs with values above limit of quantification (LOQ) detected in at least two samples in one sampling period. A carcinogenicity classification is given, if available, based on data from the International Agency for Research on Cancer (IARC, 2019). 2B=possibly carcinogenic to humans.

Across all time points, significantly higher total amounts of low molecular weight (LMW) PAHs were detected than high molecular weight (HMW) PAHs. There is a decrease in the concentration of all PAHs detected in the samples from the 1920s to the 1970s (from 709.6–216.2 ng g^-1^) apart from the 1960s which had a higher total concentration of PAHs than the 1950s with 428.0 ng g^-1^ and 367.3 ng g^-1^, respectively. The 1920s had highest average concentrations of both total LMW and total HMW PAHs, with 709.6 ng g^-1^ (351.8–1445.9 ng g^-1^) and 71.9 ng g^-1^ (19.8–110.9 ng g^-1^), respectively.

**Figure 2:**
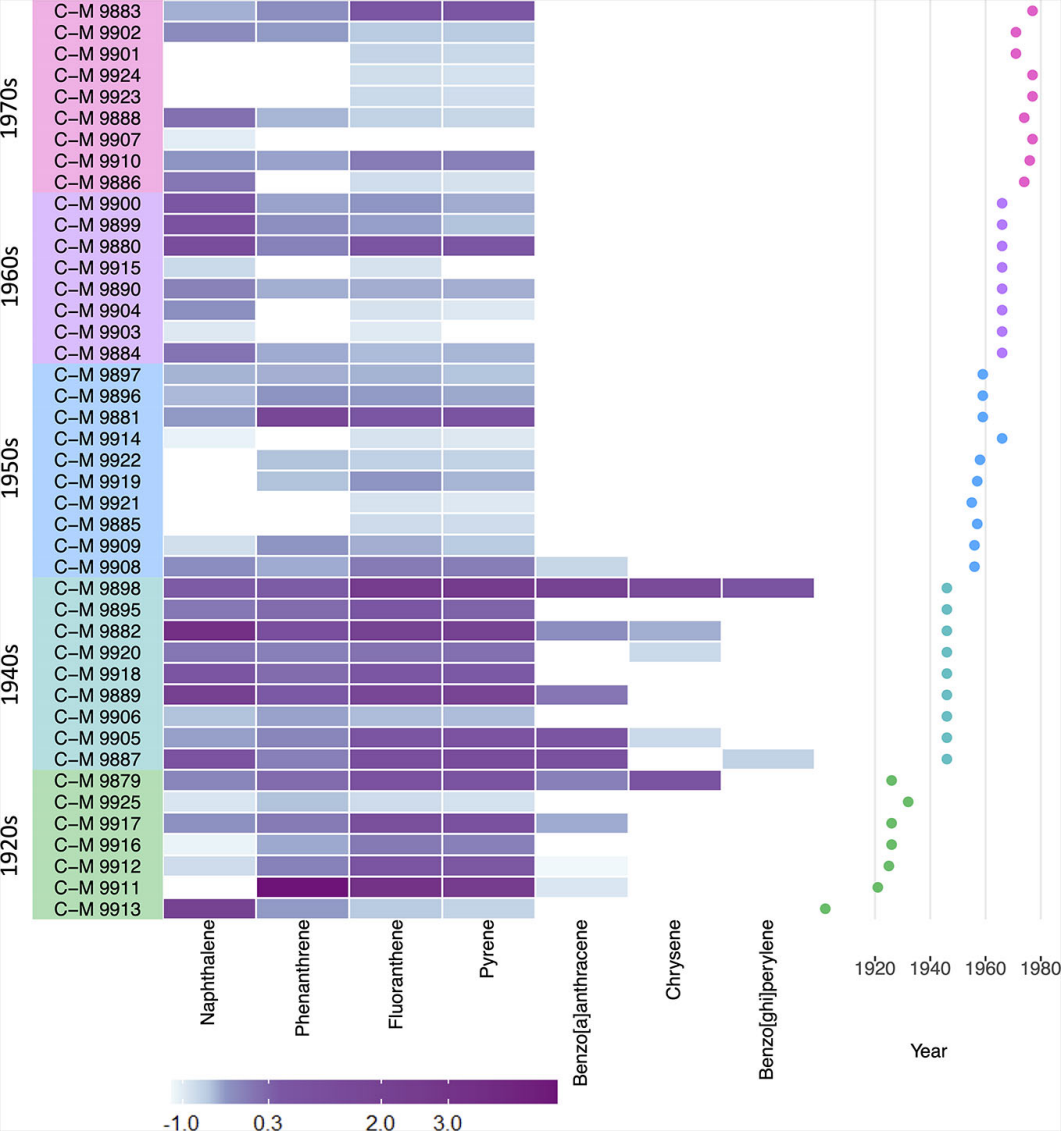
Heat map of detected PAHs across all samples divided by time period in which they were collected (1920s–1970s). The concentrations are normalized and assigned a score (column Z-score) and corresponding color, with blue being the lowest values and purple the highest amounts of compound detected in the sample. The Z-score is calculated based on the number of standard deviations that the value of the sample is above or below the mean.

**Figure 3:**
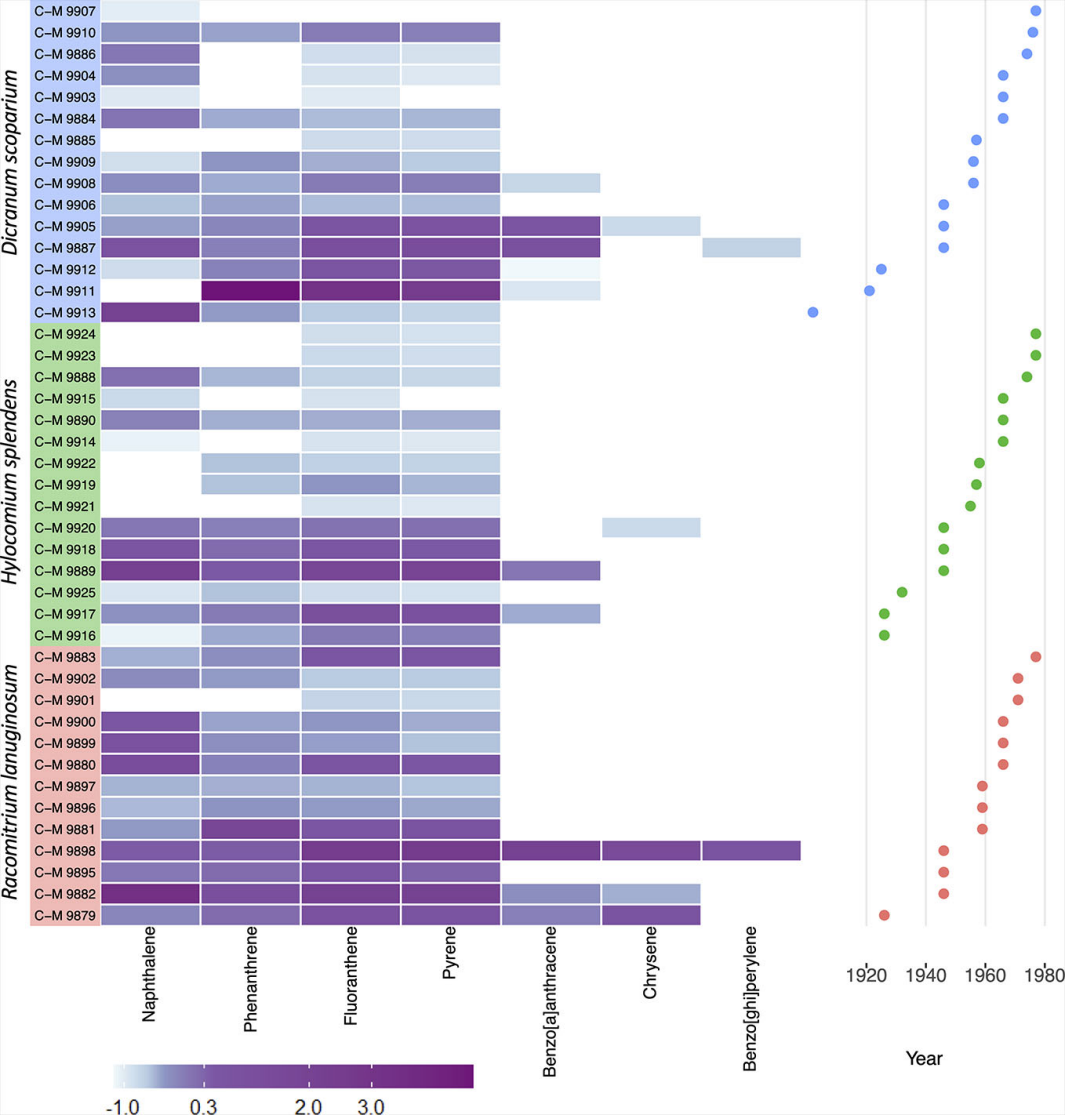
Heat map of detected PAHs and across all samples divided by species. The concentrations are normalized and assigned a score (column Z-score) and corresponding color, with blue being the lowest values and purple the highest amounts of compound detected in the sample. The Z-score is calculated based on the number of standard deviations that the value of the sample is above or below the mean.

A heat map of the data (**Figure 2**) across all three species illustrates the abundance and higher concentrations of compounds detected in samples in the 1920s and 1940s compared to the subsequent decades. In **Figure 3**, when comparing the concentrations found in the different samples across the three different species, it can be seen that there was a higher number of compounds and in higher concentrations in the samples of *D. scoparium* compared to the other species.

**Figure 4:**
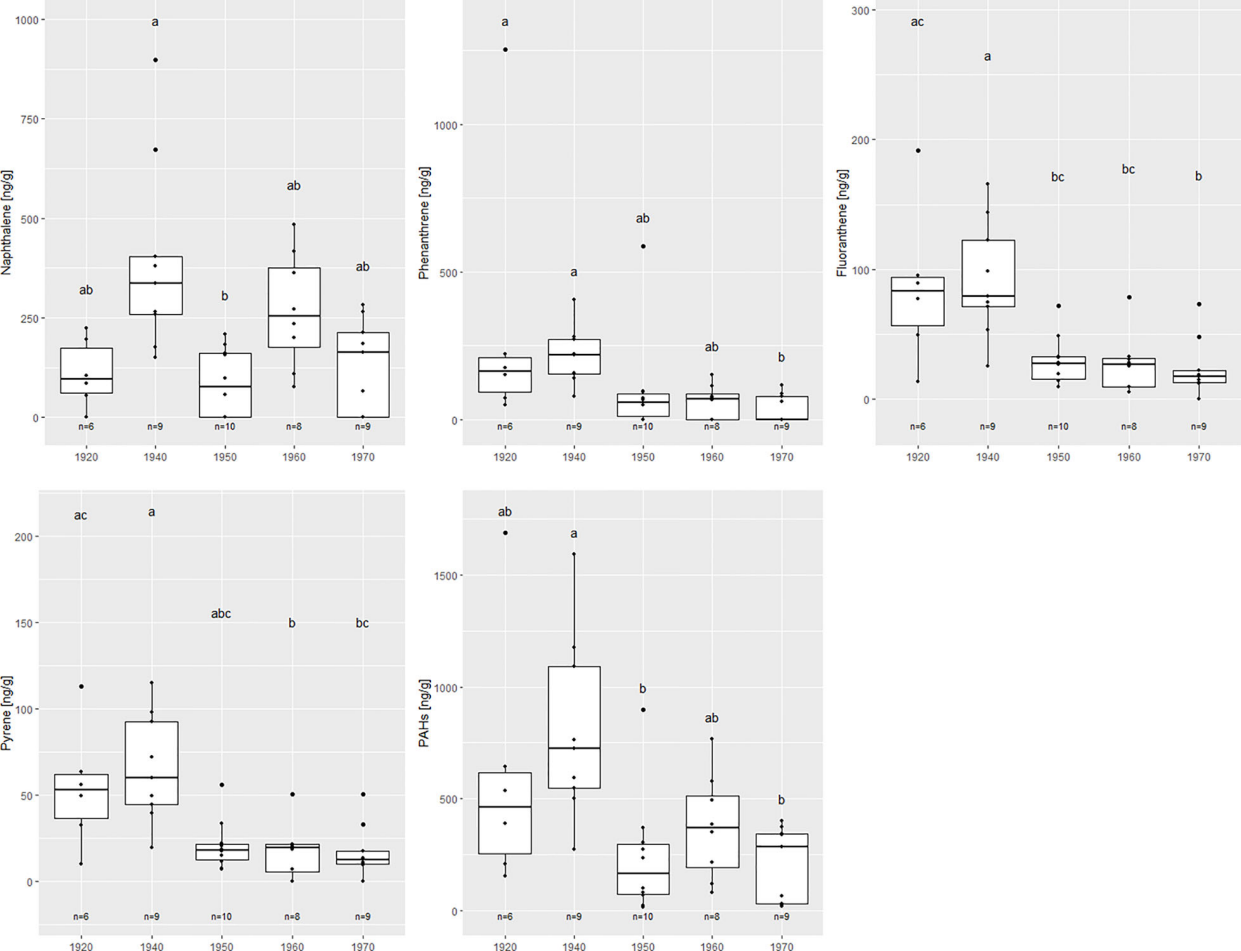
Differences in amounts of PAHs found between sampling time periods for the main compounds where significant differences were found (p<0.05).

**Figure 4** illustrates differences in the concentration of those compounds where significant differences were observed. Naphthalene was observed to increase in concentration from below 150 ng g^-1^ in the 1920s to above 250 ng g^-1^ in the 1940s (p<0.1). It then falls and peaks again in the 1960s before decreasing in concentration. The concentrations of phenanthrene and fluoranthene were significantly higher in the 1920s and 1940s than in the 1970s (p<0.1). When looking at pyrene, its concentration was significantly higher in the 1940s than the 1960s and 1970s (p<0.05). For all PAHs measured, the total PAH concentration was significantly higher in the 1940s than the 1950s and 1970s (p<0.05).

**Figure 5:**
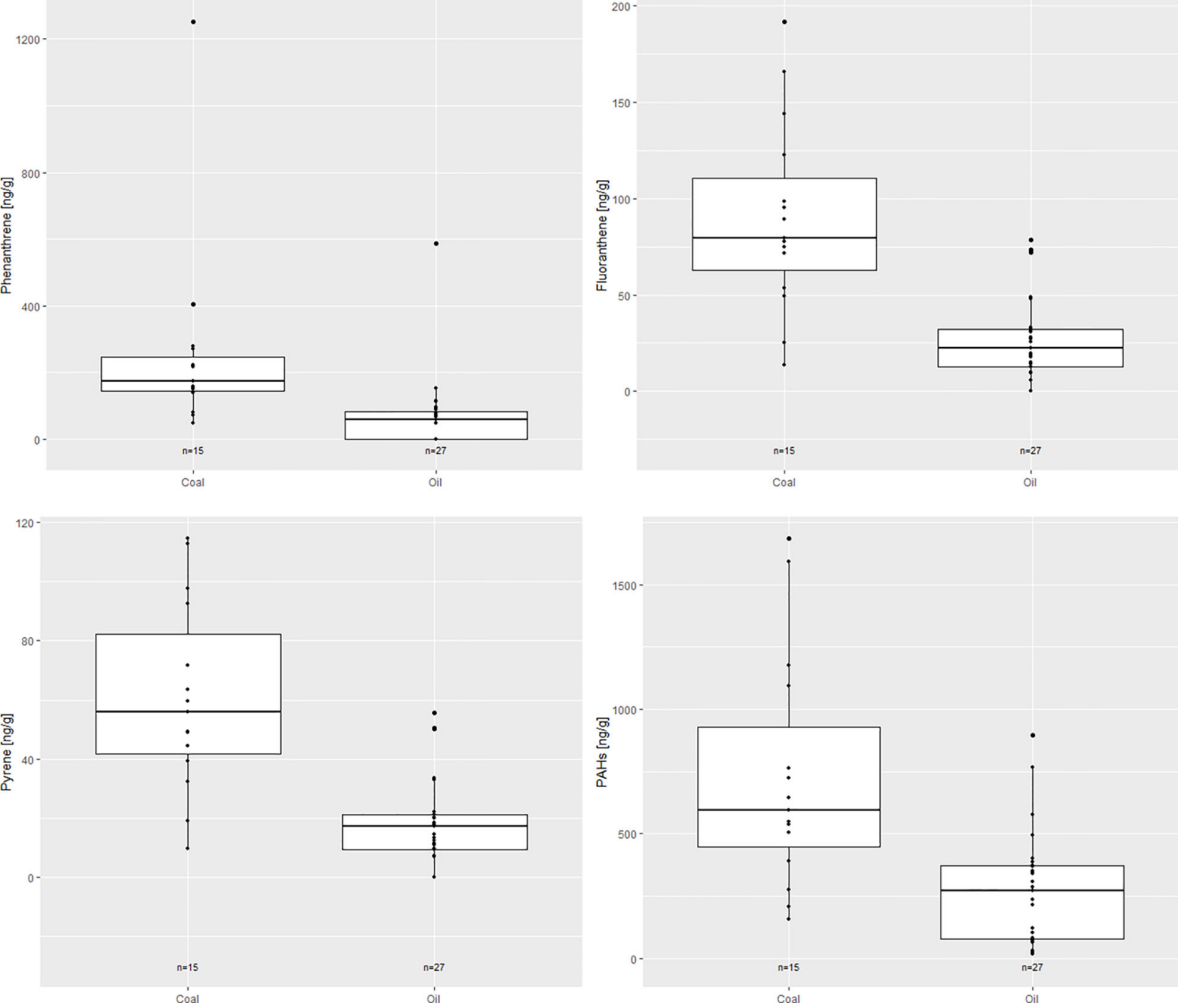
Box plot showing differences between the main source of pollution at different periods for the compounds where significant differences were found (p<0.05). Coal was the main fuel used from the 1920s–1940s and oil from the 1950s–1970s.

When samples were divided by the main source of pollution for that time (coal was the main fuel used from the 1920s–1940s and oil from the 1950s–1970s), significant differences were found for phenanthrene, fluoranthene, pyrene, and the total PAHs (**Figure 5**). Fluoranthene, pyrene, phenanthrene, and total PAHs were significantly more abundant when coal was in use (p<0.001).

To determine the sources of PAH contamination, PAH diagnostic ratios were used (Jiao et al., 2017). The ratios are listed in **Table 2** and the values calculated for the samples are listed in **Table 3**. Values for fluoranthene/(fluoranthene + pyrene) were >0.5 across all time points indicating sources from coal, wood or grass combustion. Likewise, the Fluoranthene/pyrene values were >1 pointing towards pyrogenic sources. Benz[a]anthracene/(benz[a]anthracene + chrysene) values were 0.19 for the 1920s and 0.29 for the 1950s. Values between 0.20 and 0.35 indicate PAHs from either petroleum or combustion sources. The ratio between HMW and LMW PAHs were <1 for all decades indicating PAH contamination from petrogenic sources.

**Table 2:** Diagnostic ratios used to assign the source of PAHs present in different types of samples.

PAHs	Diagnostic ratio	PAH Source
Anthracene/(Anthracene + Phenanthrene)	<0.1	Petroleum
	>0.1	Combustion
Fluoranthene/(Fluoranthene + Pyrene)	<0.4	Petroleum
	0.4-0.5	Liquid fossil fuel combustion
	>0.5	Coal, wood or grass combustion
Benz[a]anthracene/(Benz[a]anthracene + Chrysene)	<0.2	Petroleum
	0.2-0.35	Petroleum or combustion
	>0.35	Combustion
Phenanthrene/Anthracene	<10	Pyrogenic
	<15	Petrogenic
Indeno[1,2,3-cd]pyrene/Benzo[ghi]perylene	<0.2	Petrogenic
	0.2-0-5	Fuel combustion (vehicle and crude oil)
	>0.5	Coal, wood or grass combustion
Benzo[a]pyrene/(Benzo[a]pyrene + Chrysene)	<0.2	Petroleum
	0.2-0-35	Coal, wood or grass combustion
	>0.35	Fuel combustion (vehicle)
Fluoranthene/Pyrene	<1	Petrogenic
	>1	Pyrogenic
Benzo[a]pyrene/Benzo[ghi]perylene	>0.6	Fuel combustion (vehicle)
HMW/LMW PAHs	>1	Pyrogenic
	<1	Petrogenic

Table modified from Jiao et al. (2017).

**Table 3:** Diagnostic ratios used to assign the source of PAHs measured in bryophyte specimens during different sampling periods, based on the average PAH concentrations in each sampling period and the diagnostic categories listed by Jiao et al (2017).

PAHs (means & observed ranges ng g^-1^)	1920s	1940s	1950s	1960s	1970s
Fluoranthene/(Fluoranthene + Pyrene)	0.59 0.56-0.61	0.59 0.55-0.61	0.60 0.56-0.63	0.60 0.57-0.63	0.58 0.56-0.62
Benz(a)anthracene/(Benz(a)anthracene + Chrysene)	0.19 0.19-0.20	–	0.29 0.28-0.30	–	–
Fluoranthene/Pyrene	1.44 1.28-1.59	1.40 1.22-1.61	1.43 1.29-1.67	1.52 1.30-1.70	1.41 1.29-1.64
HMW/LMW PAHs	0.10	0.09	0.17	0.10	0.11
	0.05-0.14	0.03-0.80	0.03-0.71	0.07-0.18	0.06-0.78

## Discussion

To our knowledge, this study is the first study to investigate the use of mosses to measure PAHs in Greenland, but also the first to analyze plants from Greenland. Other studies carried out in Greenland have investigated U.S. EPA 16 PAHs [the 16 PAH compounds designated as priority, by the U.S. Environmental Protection Agency, see Keith (2015)] in the marine environment including mussels, with ∑PAH of 50–280 ng g^-1^ dry weight (Jörrundsdótir et al., 2014); fish with ∑PAH of 11–19 ng g^-1^ wet weight measured in fish liver (Vives et al., 2004); sediments with an average ∑PAH of 359 ng g^-1^ dry weight (Pécseli et al., 2002); and PAH deposition on snow with ∑PAH of 3,130–21,083 pg kg^-1^ water. Pécseli et al. (2002) also compared the average ∑PAH in different sample types (497 ng g^-1^ dry weight in mussels, 2,762 ng g^-1^ dry weight in fish livers and 101 ng g^-1^ dry weight in the blubber of seals). Recently, PAH levels in Crowberry (*Empetrum nigrum*) were investigated close to the town of Ilulissat in West Greenland, but the data are still unpublished. Here levels of up to 93 ng g^-1^ dry weight for single PAHs were measured. Unpublished values for soil from Qeqertarsuaq on the Disko Island reach levels of up to 1,080 ng g^-1^ dry weight for the sum of 19 PAHs including the 16 U.S. EPA PAHs. The total PAH concentrations found in this study were of the same magnitude as previous studies on mosses measuring PAHs [Japan (Oishi, 2018); France, Spain, and Switzerland (Foan et al., 2014); Spain (Foan et al., 2010); Poland (Gałuszka, 2007), Austria (Krommer et al., 2007); Hungary (Ötvös et al., 2004); China (Liu et al., 2005)]. The ∑PAH found were from 240.5 ng g^-1^ in the 1970s to 781.6 ng g^-1^ in the 1920s (**Table 1**). Like the previous studies, higher levels of naphthalene, fluoranthene, phenanthrene, and pyrene were detected in the PAH profiles, confirming these findings. The highest concentrations were recorded for naphthalene and phenanthrene, which most closely resembles the PAH profiles reported in China and Japan (Liu et al., 2005; Oishi, 2018). The ∑PAH of 240.5 ng g^-1^ in the 1970s is similar to the ∑PAH of 328.8 ng g^-1^ reported by Foan et al. (2010) for the time period 1973–1975, in their study of PAH deposition in a remote area in Spain that also included *D. scoparium*.

Overall, more LMW PAHs were detected than HMW PAHs across all time points (**Table 1**); from 6 times more in the 1950s to nearly 11 times higher in the 1940s. This is despite the fact that it has been found that mosses tend to accumulate more HMW PAHs almost to the same degree as LMW PAHs (Oishi, 2018). This is mainly due to differences in plant structure between mosses and angiosperms, which influence PAH uptake. HMW PAHs tend to be bound to particles whereas LMW PAHs are gaseous. Therefore, angiosperms favour the uptake of LMW PAHs through absorption by the stomata on their leaf surfaces or diffusion through the cuticle layer. In contrast, particle-bound HMW PAHs are more easily absorbed in mosses as they lack cuticles and they tend to uptake pollutants dissolved in precipitation as well as gaseous particles (Oishi, 2016). However, the uptake characteristics of mosses have been shown to change with climate and location. In cases where samples are far from urbanized areas, there is an increased uptake of long-range PAHs; at lower temperatures, the uptake efficiency of particle-bound PAHs is further increased, and wet deposition due to melting snow enhances absorption of long-range particle bound HMW PAHs (Oishi, 2018).

Evidence in this study strongly points to long-range atmospheric deposition of PAHs in Greenland, rather than local sources of contamination. The LMW PAHs detected in this study are largely released in a gaseous form and are therefore more likely to reach the arctic region than HMW PAHs, as has been studied for chlorinated compounds (Wania, 2003) and indicated for PAHs (Halsall et al., 2001; Lammel, 2015). The presence of more LMW PAHs than HMW PAHs is therefore evidence for long-range transport rather than local contamination sources. Local sources cannot be excluded, but the locality information provided on the herbarium samples is too vague to form a conclusion on this. With mosses dominating the Greenlandic flora, the combined conditions and features of these plants make them highly useful as bioindicators to investigate PAH atmospheric deposition in Greenland.

The PAH diagnostic ratios point to sources of PAH contamination from both petrogenic and pyrogenic sources. Furthermore, concentrations of phenanthrene, fluoranthene, naphthalene, and pyrene were quite high in the samples compared to the other PAHs. Between the 1920s and the 1970s, a decreasing trend was seen (p<0.01, **Figure 4**). These PAHs are primarily emitted as gases and are associated with wood and fossil-fuel combustion (**Figure 5**; Masclet et al., 1986; Slater et al., 2002). Their decrease coincides with the disappearance of charcoal pits and foundries in Europe and North America, and a shift away from domestic heating with wood during the 19^th^ century (**Figure 5**, Foan et al., 2010). Interestingly, there is a significant increase in total PAHs from the 1950s to 1960s (p<0.01), before they decrease again in the 1970s (**Figure 4**), due to the higher concentrations of LMWs in the 1960s (**Table 1**). Asia is thought to be a big source of the atmospheric PAHs found in Greenland (Schüpbach et al., 2018) and this peak coincides with economic development in China where a large amount of coal and petroleum was used to rapidly increase economic development (Guo et al., 2007).

As with most studies reliant on herbarium specimens, there was a bias in the sampling that was unavoidable due to non-systematic collecting activities over time and the subsequent availability of material. As the data relied on destructive sampling of herbarium specimens, we were restricted to using only those moss samples that were large enough to allow for the sampling of 1 g of dried plant material needed for the GC-MS analysis without significantly affecting the scientific value of the specimen. Unfortunately, bryophyte collecting in Greenland practically ceased in the 1970s and we were therefore not able to extend the study beyond this period and to the present day. The decrease in systematic collecting efforts is a general trend observed in many herbaria as fieldwork has become more limited. This lack of prioritization of continued collection activities consequently impacts opportunities for using museum collections to study current patterns.

Using herbarium specimens also entails a potential bias from contamination during storage. Both living and dead moss material can accumulate PAHs (Capozzi et al., 2017; Yakovleva et al., 2018). However, we assume that dead moss material will reach an equilibrium with the surroundings. As all samples had been stored for at least 40 years, we expect this equilibrium to be similar for all samples and would therefore have limited influence on the time trend observed in this study, but be an overall bias to the reported values.

Furthermore, as can be seen in **Figure 1A**, the majority of samples have been collected along the coast. This is primarily explained by the fact that only approximately 20% of Greenland is ice-free and this area is along the coast, providing habitats for mosses. All towns are also located along the coast, with most of the population living on the South-western coast (Statistics Greenland, 2018). Collectors have largely been active around the more populated areas, as reflected in the samples we have obtained. However, the potential effect of this bias is considered small as the total population of Greenland is only 57,000 spread across a large area, with the largest concentration being in Nuuk with 12,000 inhabitants. In 1900 the population was 12,000 in Greenland, in 1950 24,000 and in 1960s the total population in Greenland was around 30,000 (Statistics Greenland, 2018).

Following previous studies (Foan et al., 2010), data from the three moss species were pooled. Differences between species in uptake and accumulation capacity may exist (**Figure 4**), but this has not been studied. *Dicranum scoparium* is an acrocarpous plant (upright growing moss found in cushions or large tufts) that has a quite varied ecology being found in forests, grasslands, etc. The other two species are pleurocarpous mosses (prostrate growing). Of these, *Racomitrium languinosum* tends to prefer drier, open areas and is quite desiccation tolerant, whereas *Hylocomoum splendens* often grows associated with *D. scoparium* on the forest floor. It could be hypothesized that the growth form of *D. scoparium* may be linked to higher PAH levels (**Figure 3**) due to more compact stems that hold water for longer giving more time for absorption, but there is no data available to validate this.

Local factors may influence PAH emissions. For example, the cars in Greenland do not follow the EU emission regulations (European Commission, 2020). This would generally lead to a higher emission of air pollutants including PAHs, although the effect is expected to be small, as the number of cars in Greenland is very low (102 cars per 1000 people; Statistics Greenland, 2018). Likewise, the North Atlantic is an area with high emission from ship traffic (Johansson et al., 2017), which is likely impacting PAH deposition in Greenland. Greenland has vast natural resources which include rare earth metals, uranium, zinc, gold, iron ore, and possibly also oil and gas reserves (Statistics Greenland, 2018). With its main industry, fishing, failing to provide sustainable economic development, Greenland is looking to develop its mining sector and explore fossil fuel to combat economic hardships (Tiainen, 2016). Despite being one of the most climate-affected places in the world, Greenland controversially opted out of the 2016 Paris Agreement (a United Nations Framework Convention on Climate Change to reduce global warming through greenhouse gas emissions mitigation, adaptation and finance) in order to pursue the exploitation of its fossil fuel and mineral reserves (Vidal, 2016). This would inherently increase local PAH emissions making it vital to document and track changes in PAH deposition and exposure.

In conclusion, this study is the first to investigate the use of mosses to measure PAHs in Greenland and strongly points to long-range atmospheric deposition of PAHs in Greenland, rather than local sources of contamination. These results support the hypothesis that the Arctic is a zink for airborne pollutants and suggest continued monitoring using mosses as bioindicators may provide a valuable tool for continued surveillance of PAH atmospheric pollution.

Whereas modern moss samples can provide a measure of current day contamination, herbarium collections and their associated data provide a vital historical record of the world’s living organisms across time and space. The results of this study highlight the hidden depths of information that can further be extracted from specimens in these collections, representing an easily accessible historical record of environmental change including deposition of airborne pollutants as already suggested by Foan et al. (2010). We would also like to highlight the need to provide continuing support for both collections and the curatorial work that is required for their preservation. If collections are to provide the same wealth of information to future generations that we benefit from today, it is essential that collections are kept up-to-date and continually enriched.

## Data Availability Statement

All datasets presented in this study are included in the article/**Supplementary Material**.

## Author Contributions

KM-S, NR, and NL designed and conceptualized the project. EM collected the samples from the specimens together with NL and participated in the sample preparation for GC-MS. MC quantified the PAH concentrations. ME analyzed the data. JC designed and coordinated the GC-MS analysis and helped qualify and interpret the results. CL verified identification of the mosses. MP wrote the sections of the manuscript relating to mosses and their use as bioindicators. KM-S and NR drafted the manuscript. All authors contributed to the article and approved the submitted version.

## Funding

The research leading to these results has received funding from the People Programme (Marie Curie Actions) of the European Union’s Seventh Framework Programme FP7/2007-2013/under REA grant agreement n° [606895] for KM-S, ME, and NR.

## Conflict of Interest

The authors declare that the research was conducted in the absence of any commercial or financial relationships that could be construed as a potential conflict of interest.
