# Supplementary material for: Biomonitoring of Polycyclic Aromatic Hydrocarbon Deposition in Greenland Using Historical Moss Herbarium Specimens Shows a Decrease in Pollution During the 20th Century
Source: Front Plant Sci. 2020 Jul 15;11:1085. doi: 10.3389/fpls.2020.01085 (PMC7373755; doi:10.3389/fpls.2020.01085)
Supplement: Supplementary file 1 [file DataSheet_1.pdf]

Supplementary Material

Biomonitoring of polycyclic aromatic hydrocarbon deposition in Greenland using historical moss herbarium specimens shows a decrease in pollution during the 20th century

Karen Martinez-Swatson[1], Eszter Mihály[1], Christian Lange[1], Madeleine Ernst[1,2], Majbrit Dela Cruz[3], Michelle J. Price[4], Teis Nørgaard Mikkelsen[5], Jan H. Christensen[3], Nina Lundholm[1] & Nina Rønsted[1,6]*

[1] Natural History Museum of Denmark, Faculty of Science, University of Copenhagen, Øster Voldgade 5-7, DK-1350, Copenhagen K, Denmark
[2] Center for Newborn Screening, Department of Congenital Disorders, Statens Serum Institut, Copenhagen, Denmark
[3] Department of Plant and Environmental Sciences, Faculty of Science, University of Copenhagen, Thorvaldsensvej 40, DK-1871, Frederiksberg C, Denmark
[4] Conservatoire et Jardin Botaniques de la Ville de Genève, Chemin de l'Impératrice 1, CP 71 1292 Chambésy, Geneva, Switzerland
[5] Department of Environmental Engineering, Technical University of Denmark, Building 115, DK-2800 Kgs. Lyngby, Denmark
[6] National Tropical Botanical Garden, Kalaheo, Hawaii, HI-96741, USA

*** Correspondence:**
Nina Rønsted
nronsted@snm.ku.dk

1 Supplementary Data

2 Supplementary Figures and Tables

Supplementary Figure S1.

The polycyclic aromatic hydrocarbons (PAHs) investigated in this study, with their chemical structures and CAS numbers shown.

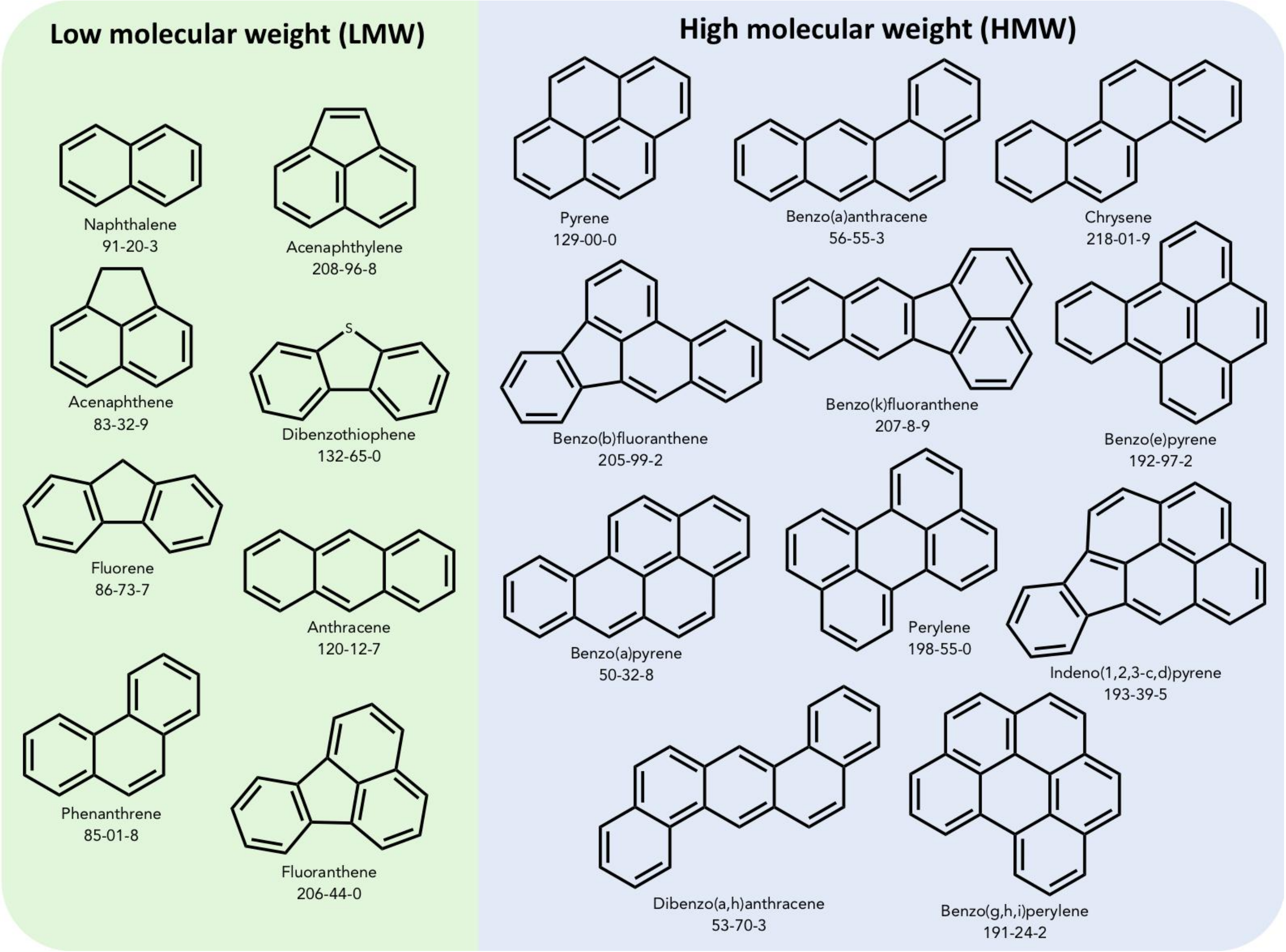

Supplementary Table S1.

List of samples used in this study with herbarium voucher information from herbarium C, Natural History Museum of Denmark, University of Copenhagen. (s.n. = sine numero/ without collection number).

Accession No.	*Species*	Collection Date	Voucher Information in Herbarium (C)
C-M 9879	*Dicranum scoparium*	10.08.1926	Igaliko; Kn. Jessen s.n.
C-M 9880	*Dicranum scoparium*	18.07.1966	Pr. Chr. Sund: Igdlorssuit; Palle Gravesen & Carlo Hansen 66-821
C-M 9881	*Dicranum scoparium*	07.1959	Qingua Dal (Julianehåb district); C. A. Jørgensen s.n.
C-M 9882	*Dicranum scoparium*	27.09.1946	Ivigtut, Nordlandet; Kjeld Holmen 5311
C-M 9883	*Dicranum scoparium*	01.08.1977	Disko: Godhavn; G. S. Mogensen & G. R. Brassard 77-159
C-M 9884	*Racomitrium lanuginosum*	27.07.1966	Eggers Ø: Itivdleq; Palle Gravesen & Carlo Hansen 66-374
C-M 9885	*Racomitrium lanuginosum*	15.06.1957	Kuanit, Arsuk; Simon Lægaard s.n.
C-M 9886	*Racomitrium lanuginosum*	03.07.1974	Umanarssûp tunua; Kjeld Holmen 74-896
C-M 9887	*Racomitrium lanuginosum*	15.09.1946	Ivigtut; Kjeld Holmen 5099
C-M 9888	*Hylocomium splendens*	23.06.1974	Head of Amitsuarssuk Fjord; Kjeld Holmen 74-233
C-M 9889	*Hylocomium splendens*	16.09.1946	Ivigtut; Kjeld Holmen 5114
C-M 9890	*Hylocomium splendens*	18.07.1966	Pr. Chr. Sund: Igdlorssuit; Palle Gravesen & Carlo Hansen 66-822
C-M 9895	*Dicranum scoparium*	13.07.1946	Tunugdliarfik; Kjeld Holmen 2017
C-M 9896	*Dicranum scoparium*	30.06.1959	Dyrnæs north of Narssaq; Else Ellitsgård s.n.
C-M 9897	*Dicranum scoparium*	24.06.1959	Dyrnæs north of Narssaq; Else Ellitsgård s.n.
C-M 9898	*Dicranum scoparium*	01.09.1946	Kangamiut; Kjeld Holmen 5012f2
C-M 9899	*Dicranum scoparium*	02.08.1966	ivssortussut; Palle Gravesen & Carlo Hansen 66-240
C-M 9900	*Dicranum scoparium*	11.07.1966	Kangerluk; Palle Gravesen & Carlo Hansen 66-753
C-M 9901	*Dicranum scoparium*	22.08.1971	Angmagssalik district: Isertoq; Jette Lewinsky 71-955
C-M 9902	*Dicranum scoparium*	24.07.1971	Fiskenæsset district: Ouvnigdlip; K. Damsholt 71-984
C-M 9903	*Racomitrium lanuginosum*	11.07.1966	Kangerdluk; Palle Gravesen & Carlo Hansen 66-711
C-M 9904	*Racomitrium lanuginosum*	27.07.1966	Eggers Ø: Itivdleq; Palle Gravesen & Carlo Hansen 66-403
C-M 9905	*Racomitrium lanuginosum*	1946	Godthaab; Kjeld Holmen 3030
C-M 9906	*Racomitrium lanuginosum*	18.06.1947	Færingerhavn; K. Jacobsen 127
C-M 9907	*Racomitrium lanuginosum*	1977	E of Sdr. Stømfjord Airport, app. 7km NW of Kegen; G. S. Mogensen & G. R. Brassard 77-047
C-M 9908	*Racomitrium lanuginosum*	20.06.1956	Disko Fjord: Kangerdluarssuk; Kjeld Holmen 14.109
C-M 9909	*Racomitrium lanuginosum*	27.08.1956	Nugssuaq Pen.: Agatdalen; Kjeld Holmen 13.033
C-M 9910	*Racomitrium lanuginosum*	01.09.1976	Upernavik; Vibeke Sünksen 56
C-M 9911	*Racomitrium lanuginosum*	09.08.1921	Igdloluarssuit; J. Noe-Nygaard s.n.
C-M 9912	*Racomitrium lanuginosum*	14.08.1925	Narssalik; A. E. Porsild & M. P. Porsild s.n.
C-M 9914	*Hylocomium splendens*	15.07.1966	Kangerdluk; Palle Gravesen & Carlo Hansen 66-685
C-M 9915	*Hylocomium splendens*	05.07.1966	Kangerssuneq qingordleq: Ánivia; Palle Gravesen & Carlo Hansen 66-422
C-M 9916	*Hylocomium splendens*	04.09.1926	Akuliarusek. E. For Igalikofjord; Kn. Jessen s.n.
C-M 9917	*Hylocomium splendens*	11.09.1926	Sioralik, innermost part of Agdluitsok; Kn. Jessen s.n.
C-M 9918	*Hylocomium splendens*	09.07.1946	N. Grønne Dal.; Kjeld Holmen 1325
C-M 9919	*Hylocomium splendens*	24.08.1957	ca. 10 km. east of Arsuk; Simon Lægaard s.n.
C-M 9920	*Hylocomium splendens*	19.07.1946	Godthaab; Kjeld Holmen 3057
C-M 9921	*Hylocomium splendens*	16.06.1955	Frederikshaab; Eva Clausen A.26
C-M 9922	*Hylocomium splendens*	06.07.1958	Between the bay of Muslingebugten, at the entrance of Evighedsfjord, and a small lake 1/2 km farther east; Tyge Christensen s.n.
C-M 9923	*Hylocomium splendens*	06.08.1977	Sukkertoppen district, Qivâqe; Marienne Hansen 507
C-M 9924	*Hylocomium splendens*	28.07.1977	Sdr. Stømfjord, Airport - area; G. S. Mogensen & G. R. Brassard 77-005
C-M 9925	*Hylocomium splendens*	12.08.1932	Seqineqarajutoq at Orpigsoq; L. Harmsen 26

Supplementary Table S2. List of internal and recovery standards used for the quantification of PAHs and alkylated (alkyl) PAHs.

PAH	Internal standard	Recovery standard
Naphthalene, Acenaphthylene	Naphthalene-d8	Acenaphthylene-d8
Acenaphthene	Acenaphthene-d10	Anthracene-d10
Fluorene	Fluorene-d10	Anthracene-d10
Dibenzothiophene	Dibenzothiophene-d8	Anthracene-d10
Anthracene, phenanthrene	Phenanthrene-d10	Anthracene-d10
Pyrene, fluoranthene	Pyrene-d10	Fluoranthene-d10
Chrysene, benzo[a]anthracene	Chrysene-d12	Benzo[a]anthracene-d12
Benzo[b]fluoranthene, benzo[k]fluoranthene, benzo[e]pyrene, benzo[a]pyrene, perylene	Benzo[k]fluoranthene-d12	Benzo[a]pyrene-d12
Dibenzo[ah]anthracene, b enzo[ghi]perylene, indeno[1,2,3-cd]pyrene	Benzo[ghi]perylene-d12	Indeno[1,2,3-cd]pyrene-d12

Supplementary Table S3. Detection limit (DL) and limit of quantification (LOQ) of PAHs measured in moss samples by GC-MS; given in ng g^{-1} dry weight. Values are based on 100% recovery and an estimation of the standard deviation of the lowest standard based on the standard deviation of the highest standard that was run four times.

PAH	DL (ng g^{-1})	LOQ (ng g^{-1})
**Naphthalene**	26.4	55.6
**Acenaphthylene**	7.5	24.4
**Acenaphthene**	24.0	75.1
**Fluorene**	10.4	31.5
**Dibenzothiophene**	15.6	51.3
**Phenanthrene**	17.0	39.3
**Anthracene**	2.5	8.1
**Fluoranthene**	2.8	5.4
**Pyrene**	2.2	5.7
**Benzo[a]anthracene**	2.0	6.7
**Chrysene**	11.9	38.9
**Benzo[b]fluoranthene**	14.2	42.0
**Benzo[k]fluoranthene**	14.1	46.7
**Benzo[e]pyrene**	11.7	39.0
**Benzo[a]pyrene**	11.3	36.5
**Perylene**	8.4	28.0
**Indeno[1,2,3-cd]pyrene**	14.2	47.0
**Dibenzo[ah]anthracene**	8.2	27.0
**Benzo[ghi]perylene**	6.4	16.1

Supplementary Table S4. Concentration of PAHs in ng g^{-1} dry weight measured in bryophyte samples by GC-MS (<LOQ = below limit of quantification).

Sample	Species	Collection Year	Naphthalene	Acenaphthylene	Acenaphthene	Fluorene	Dibenzothiophene	Phenanthrene	Anthracene	Fluoranthene	Pyrene	Benzo[a]anthracene	Chrysene	Benzo[b]fluoranthene	Benzo[k]fluoranthene	Benzo[e]pyrene	Benzo[a]pyrene	Perylene	Indeno[1,2,3-cd]pyrene	Dibenzo[ah]anthracene	Benzo[ghi]perylene
C-M 9879	*Dicranum scoparium*	1926	224.4	<LOQ	<LOQ	<LOQ	<LOQ	221.4	<LOQ	89.1	56.0	10.2	44.8	<LOQ	<LOQ	<LOQ	<LOQ	<LOQ	<LOQ	<LOQ	<LOQ
C-M 9880	*Dicranum scoparium*	1966	484.9	<LOQ	<LOQ	<LOQ	<LOQ	152.1	<LOQ	78.4	50.5	<LOQ	<LOQ	<LOQ	<LOQ	<LOQ	<LOQ	<LOQ	<LOQ	<LOQ	<LOQ
C-M 9881	*Dicranum scoparium*	1959	183.0	<LOQ	<LOQ	<LOQ	<LOQ	586.8	<LOQ	72.2	55.6	<LOQ	<LOQ	<LOQ	<LOQ	<LOQ	<LOQ	<LOQ	<LOQ	<LOQ	<LOQ
C-M 9882	*Dicranum scoparium*	1946	897.0	<LOQ	<LOQ	<LOQ	<LOQ	405.1	<LOQ	143.8	97.7	9.4	37.3	<LOQ	<LOQ	<LOQ	<LOQ	<LOQ	<LOQ	<LOQ	<LOQ
C-M 9883	*Dicranum scoparium*	1977	163.9	<LOQ	<LOQ	<LOQ	<LOQ	114.8	<LOQ	73.2	50.1	<LOQ	<LOQ	<LOQ	<LOQ	<LOQ	<LOQ	<LOQ	<LOQ	<LOQ	<LOQ
C-M 9884	*Racomitrium lanuginosum*	1966	270.9	<LOQ	<LOQ	<LOQ	<LOQ	71.5	<LOQ	25.5	19.8	<LOQ	<LOQ	<LOQ	<LOQ	<LOQ	<LOQ	<LOQ	<LOQ	<LOQ	<LOQ
C-M 9885	*Racomitrium lanuginosum*	1957	<LOQ	<LOQ	<LOQ	<LOQ	<LOQ	<LOQ	<LOQ	13.9	11.4	<LOQ	<LOQ	<LOQ	<LOQ	<LOQ	<LOQ	<LOQ	<LOQ	<LOQ	<LOQ
C-M 9886	*Racomitrium lanuginosum*	1974	264.2	<LOQ	<LOQ	<LOQ	<LOQ	<LOQ	<LOQ	12.7	9.2	<LOQ	<LOQ	<LOQ	<LOQ	<LOQ	<LOQ	<LOQ	<LOQ	<LOQ	<LOQ
C-M 9887	*Racomitrium lanuginosum*	1946	403.1	<LOQ	<LOQ	<LOQ	<LOQ	156.6	<LOQ	98.4	71.8	15.2	<LOQ	<LOQ	<LOQ	<LOQ	<LOQ	<LOQ	<LOQ	<LOQ	18.9
C-M 9888	*Hylocomium splendens*	1974	281.4	<LOQ	<LOQ	<LOQ	<LOQ	59.6	<LOQ	18.8	13.3	<LOQ	<LOQ	<LOQ	<LOQ	<LOQ	<LOQ	<LOQ	<LOQ	<LOQ	<LOQ
C-M 9889	*Hylocomium splendens*	1946	671.8	<LOQ	<LOQ	<LOQ	<LOQ	278.9	<LOQ	122.4	92.4	10.9	<LOQ	<LOQ	<LOQ	<LOQ	<LOQ	<LOQ	<LOQ	<LOQ	<LOQ
C-M 9890	*Hylocomium splendens*	1966	235.6	<LOQ	<LOQ	<LOQ	<LOQ	66.6	<LOQ	28.2	21.0	<LOQ	<LOQ	<LOQ	<LOQ	<LOQ	<LOQ	<LOQ	<LOQ	<LOQ	<LOQ
C-M 9895	*Dicranum scoparium*	1946	259.1	<LOQ	<LOQ	<LOQ	<LOQ	218.2	<LOQ	71.6	44.4	<LOQ	<LOQ	<LOQ	<LOQ	<LOQ	<LOQ	<LOQ	<LOQ	<LOQ	<LOQ
C-M 9896	*Dicranum scoparium*	1959	156.3	<LOQ	<LOQ	<LOQ	<LOQ	96.3	<LOQ	31.9	21.9	<LOQ	<LOQ	<LOQ	<LOQ	<LOQ	<LOQ	<LOQ	<LOQ	<LOQ	<LOQ
C-M 9897	*Dicranum scoparium*	1959	161.5	<LOQ	<LOQ	<LOQ	<LOQ	66.1	<LOQ	27.1	18.2	<LOQ	<LOQ	<LOQ	<LOQ	<LOQ	<LOQ	<LOQ	<LOQ	<LOQ	<LOQ
C-M 9898	*Dicranum scoparium*	1946	335.6	<LOQ	<LOQ	<LOQ	<LOQ	271.7	<LOQ	165.5	114.7	20.8	49.5	46.3	50.7	<LOQ	<LOQ	<LOQ	<LOQ	<LOQ	37.2
C-M 9899	*Dicranum scoparium*	1966	417.2	<LOQ	<LOQ	<LOQ	<LOQ	111.8	<LOQ	30.8	18.5	<LOQ	<LOQ	<LOQ	<LOQ	<LOQ	<LOQ	<LOQ	<LOQ	<LOQ	<LOQ
C-M 9900	*Dicranum scoparium*	1966	361.5	<LOQ	<LOQ	<LOQ	<LOQ	77.9	<LOQ	32.8	21.2	<LOQ	<LOQ	<LOQ	<LOQ	<LOQ	<LOQ	<LOQ	<LOQ	<LOQ	<LOQ
C-M 9901	*Dicranum scoparium*	1971	<LOQ	<LOQ	<LOQ	<LOQ	<LOQ	<LOQ	<LOQ	17.7	12.5	<LOQ	<LOQ	<LOQ	<LOQ	<LOQ	<LOQ	<LOQ	<LOQ	<LOQ	<LOQ
C-M 9902	*Dicranum scoparium*	1971	212.6	<LOQ	<LOQ	<LOQ	<LOQ	87.2	<LOQ	22.4	17.3	<LOQ	<LOQ	<LOQ	<LOQ	<LOQ	<LOQ	<LOQ	<LOQ	<LOQ	<LOQ
C-M 9903	*Racomitrium lanuginosum*	1966	76.2	<LOQ	<LOQ	<LOQ	<LOQ	<LOQ	<LOQ	5.6	<LOQ	<LOQ	<LOQ	<LOQ	<LOQ	<LOQ	<LOQ	<LOQ	<LOQ	<LOQ	<LOQ
C-M 9904	*Racomitrium lanuginosum*	1966	199.3	<LOQ	<LOQ	<LOQ	<LOQ	<LOQ	<LOQ	9.8	6.8	<LOQ	<LOQ	<LOQ	<LOQ	<LOQ	<LOQ	<LOQ	<LOQ	<LOQ	<LOQ
C-M 9905	*Racomitrium lanuginosum*	1946	176.5	<LOQ	<LOQ	<LOQ	<LOQ	138.6	<LOQ	79.3	59.6	13.7	35.4	<LOQ	<LOQ	<LOQ	<LOQ	<LOQ	<LOQ	<LOQ	<LOQ
C-M 9906	*Racomitrium lanuginosum*	1947	149.9	<LOQ	<LOQ	<LOQ	<LOQ	79.2	<LOQ	25.4	19.1	<LOQ	<LOQ	<LOQ	<LOQ	<LOQ	<LOQ	<LOQ	<LOQ	<LOQ	<LOQ
C-M 9907	*Racomitrium lanuginosum*	1977	64.8	<LOQ	<LOQ	<LOQ	<LOQ	<LOQ	<LOQ	<LOQ	<LOQ	<LOQ	<LOQ	<LOQ	<LOQ	<LOQ	<LOQ	<LOQ	<LOQ	<LOQ	<LOQ
C-M 9908	*Racomitrium lanuginosum*	1956	208.0	<LOQ	<LOQ	<LOQ	<LOQ	71.4	<LOQ	48.9	33.5	7.2	<LOQ	<LOQ	<LOQ	<LOQ	<LOQ	<LOQ	<LOQ	<LOQ	<LOQ
C-M 9909	*Racomitrium lanuginosum*	1956	97.1	<LOQ	<LOQ	<LOQ	<LOQ	92.2	<LOQ	28.0	17.4	<LOQ	<LOQ	<LOQ	<LOQ	<LOQ	<LOQ	<LOQ	<LOQ	<LOQ	<LOQ
C-M 9910	*Racomitrium lanuginosum*	1976	185.9	<LOQ	<LOQ	<LOQ	<LOQ	78.6	<LOQ	48.1	32.9	<LOQ	<LOQ	<LOQ	<LOQ	<LOQ	<LOQ	<LOQ	<LOQ	<LOQ	<LOQ
C-M 9911	*Racomitrium lanuginosum*	1921	N/A	<LOQ	<LOQ	<LOQ	72.3	1252.9	50.1	191.8	112.9	6.3	<LOQ	<LOQ	<LOQ	<LOQ	<LOQ	<LOQ	<LOQ	<LOQ	<LOQ
C-M 9912	*Racomitrium lanuginosum*	1925	105.1	<LOQ	<LOQ	<LOQ	<LOQ	151.1	<LOQ	77.5	49.4	5.3	<LOQ	<LOQ	<LOQ	<LOQ	<LOQ	<LOQ	<LOQ	<LOQ	<LOQ
C-M 9914	*Hylocomium splendens*	1966	55.9	<LOQ	<LOQ	<LOQ	<LOQ	<LOQ	<LOQ	9.3	7.1	<LOQ	<LOQ	<LOQ	<LOQ	<LOQ	<LOQ	<LOQ	<LOQ	<LOQ	<LOQ

C-M 9915	*Hylocomium splendens*	1966	109.9	<LOQ	<LOQ	<LOQ	<LOQ	<LOQ	<LOQ	9.7	<LOQ	<LOQ	<LOQ	<LOQ	<LOQ	<LOQ	<LOQ	<LOQ	<LOQ	<LOQ	<LOQ
C-M 9916	*Hylocomium splendens*	1926	53.7	<LOQ	<LOQ	<LOQ	<LOQ	73.1	<LOQ	49.4	32.2	<LOQ	<LOQ	<LOQ	<LOQ	<LOQ	<LOQ	<LOQ	<LOQ	<LOQ	<LOQ
C-M 9917	*Hylocomium splendens*	1926	196.0	<LOQ	<LOQ	<LOQ	<LOQ	174.0	<LOQ	95.3	63.4	8.5	<LOQ	<LOQ	<LOQ	<LOQ	<LOQ	<LOQ	<LOQ	<LOQ	<LOQ
C-M 9918	*Hylocomium splendens*	1946	379.2	<LOQ	<LOQ	<LOQ	<LOQ	221.5	<LOQ	74.8	49.1	<LOQ	<LOQ	<LOQ	<LOQ	<LOQ	<LOQ	<LOQ	<LOQ	<LOQ	<LOQ
C-M 9919	*Hylocomium splendens*	1957	<LOQ	<LOQ	<LOQ	<LOQ	<LOQ	48.2	<LOQ	33.0	20.1	<LOQ	<LOQ	<LOQ	<LOQ	<LOQ	<LOQ	<LOQ	<LOQ	<LOQ	<LOQ
C-M 9920	*Hylocomium splendens*	1946	265.2	<LOQ	<LOQ	<LOQ	<LOQ	155.4	<LOQ	53.5	39.2	<LOQ	35.4	<LOQ	<LOQ	<LOQ	<LOQ	<LOQ	<LOQ	<LOQ	<LOQ
C-M 9921	*Hylocomium splendens*	1955	<LOQ	<LOQ	<LOQ	<LOQ	<LOQ	<LOQ	<LOQ	9.3	7.0	<LOQ	<LOQ	<LOQ	<LOQ	<LOQ	<LOQ	<LOQ	<LOQ	<LOQ	<LOQ
C-M 9922	*Hylocomium splendens*	1958	<LOQ	<LOQ	<LOQ	<LOQ	<LOQ	48.1	<LOQ	19.5	14.6	<LOQ	<LOQ	<LOQ	<LOQ	<LOQ	<LOQ	<LOQ	<LOQ	<LOQ	<LOQ
C-M 9923	*Hylocomium splendens*	1977	<LOQ	<DL	<LOQ	<LOQ	<LOQ	<LOQ	<LOQ	14.9	11.0	<LOQ	<LOQ	<LOQ	<DL	<LOQ	<LOQ	<LOQ	<LOQ	<LOQ	<LOQ
C-M 9924	*Hylocomium splendens*	1977	<LOQ	<LOQ	<LOQ	<LOQ	<LOQ	<LOQ	<LOQ	12.5	9.7	<LOQ	<LOQ	<LOQ	<LOQ	<LOQ	<LOQ	<LOQ	<LOQ	<LOQ	<LOQ
C-M 9925	*Hylocomium splendens*	1932	84.7	<LOQ	<LOQ	<LOQ	<LOQ	48.1	<LOQ	13.4	9.7	<LOQ	<LOQ	<LOQ	<LOQ	<LOQ	<LOQ	<LOQ	<LOQ	<LOQ	<LOQ
